# JCPDS-ICDD Research Associateship (Cooperative Program with NBS/NIST)

**DOI:** 10.6028/jres.106.052

**Published:** 2001-12-01

**Authors:** W. Wong-Ng, H. F. McMurdie, C. R. Hubbard, A. D. Mighell

**Affiliations:** National Institute of Standards and Technology, Gaithersburg, MD 20899-0001

**Keywords:** CD-ROM data storage, powder data evaluation, Research Associateship, SRMs for powder diffraction, standard powder x-ray diffraction patterns

## Abstract

The Research Associateship program of the Joint Committee on Powder Diffraction-International Centre for Diffraction Data (JCPDS-ICDD, now known as the ICDD) at NBS/NIST was a long standing (over 35 years) successful industry-government cooperation. The main mission of the Associateship was to publish high quality x-ray reference patterns to be included in the Powder Diffraction File (PDF). The PDF is a continuing compilation of patterns gathered from many sources, compiled and published by the ICDD. As a result of this collaboration, more than 1500 high quality powder diffraction patterns, which have had a significant impact on the scientific community, were reported. In addition, various research collaborations with NBS/NIST also led to the development of several standard reference materials (SRMs) for instrument calibration and quantitative analyses, and computer software for data collection, calibration, reduction, for the editorial process of powder pattern publication, analysis of powder data, and for quantitative analyses. This article summarizes information concerning the JCPDS-ICDD organization, the Powder Diffraction File (PDF), history and accomplishments of the JCPDS-ICDD Research Associateship.

## 1. Early History

### 1.1 Powder X-Ray Diffraction Patterns

In the late thirties, the powder x-ray diffraction technique was recognized as a powerful technique for phase identification/chemical analysis. In 1938, Hanawalt, Rinn, and Frevel [1] were the first to report tabulated data on diffraction patterns of 1000 chemical substances; they also gave a scheme of classification which made possible a routine and valuable use in the chemical laboratory of the Hull method of x-ray analysis which was published in 1919 [2]. Hull stated “That every crystalline substance gives a pattern; that the same substance always gives the same pattern; and that in a mixture of substances each produces its pattern independently of the others, so that the photograph obtained with a mixture is the superimposed sum of the photographs that would be obtained by exposing each of the components separately for the same length of time. This law applies quantitatively to the intensities of the lines, as well as to their positions, so that the method is capable of development as a quantitative analysis.” This scheme of classification by Hanawalt et al. [1] using the three strongest peaks of a pattern for identification of unknowns was later called the “Hanawalt Search”, a tribute to his profound contribution to the powder diffraction field in the years to come.

### 1.2 Formation of the JCPDS

In the late 1930s, a meeting was held at Gibson Island, MD to discuss the formation of an ongoing file of x-ray powder diffraction patterns of chemical phases for use in identifying materials. This meeting was arranged by Wheeler P. Davey of Pennsylvania State College. Herbert Insley and Howard McMurdie were invited to the meeting as NBS was known to have a growing interest in the subject. In 1941, the Joint Committee on Chemical Analysis by X-Ray Diffraction Methods, part of the American Society for Testing and Materials (ASTM), was founded. This committee, which was also sponsored by the British Institute of Physics, asked Davey to direct an extensive project to coordinate research and publication activity of the diffraction patterns worldwide [3,4]. This activity called upon numerous leaders in the x-ray diffraction field to categorize a collection of cards with information pertaining to powder x-ray diffraction patterns. Howard McMurdie was asked to represent NBS. For several years the size of the committee expanded, and its members met at Pennsylvania State University on a regular basis. Later the chairman for the committee was J. V. Smith, also from Penn State.

ASTM published the first set of data, consisting of 4000 cards in 1941, covering some 1300 compounds. An extensive set of some 4500 supplement cards was completed around 1945. These cards contained data for inorganic, organic and mineral crystalline phases and included interplanar spacings, relative intensities, and, when available, indexing, unit cell, specific gravity, and optical property data.

The purpose of this committee was to collect, edit, publish, and distribute primary references for x-ray powder diffraction analysis/identification of crystalline materials. This compilation is now commonly known as the Powder Diffraction File™ (PDF). Many scientific associations and societies, including the ASTM, supported the initial effort. Continued additions and regular publication of “sets” followed. By 1969, the committee was constituted as a Pennsylvania nonprofit corporation under the title of the Joint Committee on Powder Diffraction Standards (JCPDS). In 1978, the current name—International Centre for Diffraction Data—was adopted. The ICDD Mission Statement is “The International Centre for Diffraction Data will continue as the world center for quality diffraction data to meet the needs of the technical community, ICDD promotes the applications of materials characterization methods in science and technology by providing a forum for the exchange of ideas and information.”

Today, nearly 300 international scientists from industrial, academic and governmental laboratories comprise the active membership from which the organization draws its Board of Directors, committees, and subcommittees. The members, who are volunteers, are actively engaged in developing the field of x-ray powder diffraction and related disciplines. They gather twice annually to discuss various technical issues related to powder diffraction methods, and editorial issues related to the PDF, and to organize, plan, and review policies and procedures within the ICDD organization. The Technical Committee consists of three categories of subcommittees, namely, Materials Subcommittee (Ceramics, Minerals, Metals and Alloys, Organic and Pharmaceutical and Polymer), Characterization Methods & Tools Subcommittee (Electron Diffraction, Synchrotron Diffraction Methods, X-Ray Diffraction Methods, X-Ray Fluorescence, High Pressure and High Temperature Diffraction, and Neutron Powder Diffraction), and ICDD Activities Subcommittee (Database, Education and PDF Editorial Staff). A paid scientific and administrative staff, located at the headquarters in Pennsylvania, is responsible for the production of the various databases offered by the ICDD.

The ICDD financially supports scientific activities worldwide. Amongst these activities, it maintains a Grants-in-Aid program in order to provide high quality data and develop search techniques for the identification of materials by powder diffraction methods. In the area of education, the ICDD “has established a Crystallography Scholarship Fund, now known as the Ludo Frevel Crystallography Scholarship Fund.” The function of this fund is “to encourage promising graduate students to pursue crystallographically oriented research.”

### 1.3 Formation of the JCPDS-Associateship at NBS

In the late forties, there was considerable criticism about duplicate patterns on some phases which were not always consistent as they were made by different methods, and there was always a call for coverage of more compounds. For example, at a meeting of the American Crystallographic Society in 1948 a prominent crystallographer called for the whole file to be redone. It was recognized that there was a need for an organized scientific group to undertake a systematic review of the file, and to prepare quality powder diffraction reference standards. Although many of the patterns obtained from the literature and other sources were adequate, there was a need was to prepare data using reproducible, controlled conditions of specimen preparation and the best recording instrumentation available. Under the sponsorship of the ASTM, the Joint Committee on Chemical Analysis by X-Ray Diffraction Methods of the ASTM, A.S.X.-R.E.D., and British Institute of Physics, a Research Associateship was established at NBS, effective July 18, 1949 [4].

The Research Associateship was under the direct supervision of H. F. McMurdie, then Chief of the Constitution and Microstructure Section of the Division of Mineral products, and members of this special group were appointed by the JCPDS-ICDD, consisting of Chairman W. L. Fink (Aluminum Company of America), L. L. Wyman (General Electric Co.), W. P. Davey, and L. K. Frevel (Dow Chemical Co.). Eleanor Tatge was named to the Associateship. Her primary responsibility was to review conflicting data in the card file issued by the Joint Committee and undertake the selection of correct patterns or to make new patterns of standard material After McMurdie’s retirement, he was appointed by the JCPDS-ICDD to be the main contact with ICDD and H. Swanson became the principal scientist. In 1976 Marlene Morris became the contact and principal scientist. In the early seventies, Camden Hubbard of NBS was appointed as the liaison officer between the Associateship and NBS. He provided distinguished leadership in ensuring the quality of the patterns, as well as keeping abreast with the state-of-the-art computer technology and x-ray diffraction instrumentation and techniques.

**Fig. 1:**
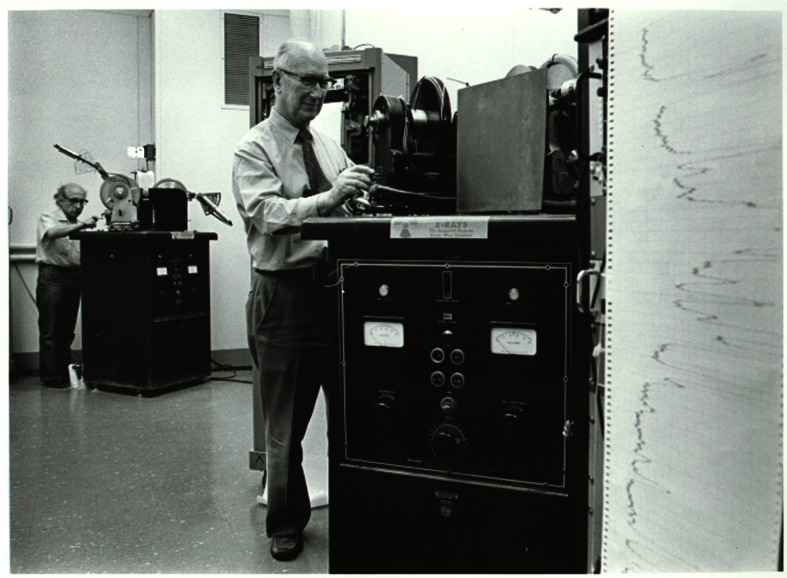
Photo of the early diffractometers (seventies and early eighties) and strip-chart recorders used to collect powder diffraction patterns. Howard McMurdie is shown in the front and Boris Paretzkin (deceased) in the back.

**Fig. 2:**
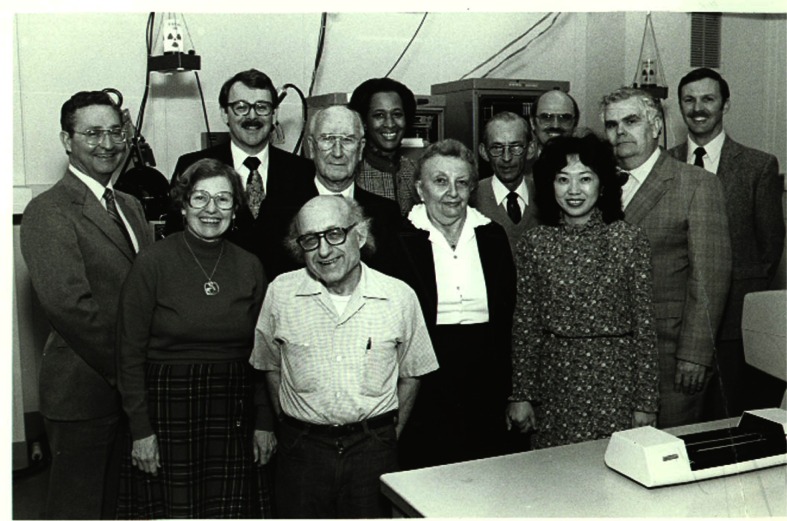
Members of the JCPDS Associateship with members of the JCPDS Board of Directors. (From left to right, front row: Eloise Evans, Boris Paretzkin, Mary Mrose, Winnie Wong-Ng; back row: Julian Messick (general manager of JCPDS), James Edmond (JCPDS member of BOD), Howard McMurdie, Marlene Morris, Harry Parker, Gregory McCarthy (JCPDS member of BOD), Deane Smith (JCPDS Chairman of BOD), and Camden Hubbard (NBS/JCPDS Liaison).

**Fig. 3:**
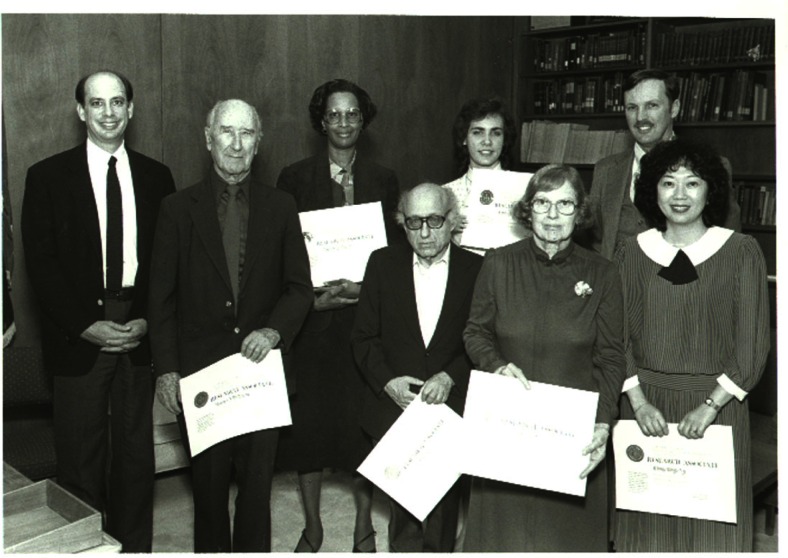
In 1986, the mission of the JCPDS/NBS collaboration was accomplished. This photo shows the Associateship members receiving a certificate of accomplishment from the NBS Deputy Director (later Director), Ray Kammer. From left to right, front row: Howard McMurdie, Boris Paretzkin, Eloise Evans, Winnie Wong-Ng; back row: Ray Kammer, Marlene Morris, Kimberly Kessell (Hill), and Camden Hubbard.

**Fig. 4:**
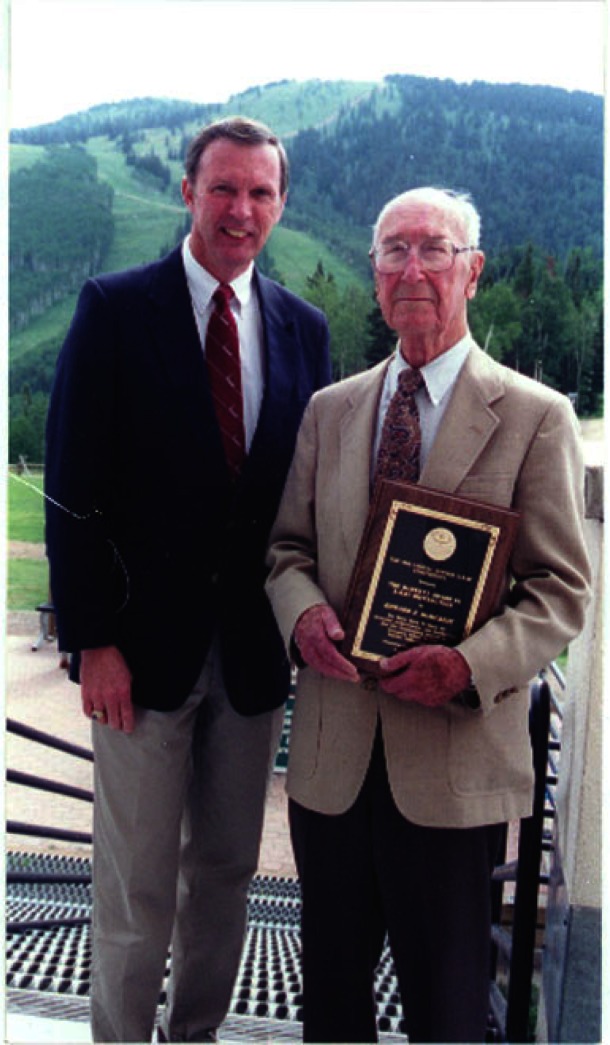
Howard McMurdie receiving the 2000 Barrett Award from the Chair of the Board of Director, Camden Hubbard.

The members of the Associateship from the beginning to its conclusion in 1986, included (alphabetical order): Simon J. Carmel, Eloise H. Evans, Ruth K. Fuyat, Nancy T. Gilfrich, Donna M. Gladhill, Johan H. de Groot, Kimberly Hill, Camden R. Hubbard, Howard F. McMurdie, Marlene C. Morris (Cook), Boris Paretzkin, Harry S. Parker, Nikos P. Pyrros, Eleanor Tatge, Roger P. Stinchfield, Howard E. Swanson, George M. Ugrinic, Linda Ulmer, and Winnie Wong-Ng. A few photographs of these members and outside colleagues are shown in Figs. 1 to 4.

## 2. The Powder Diffraction File (PDF)

In the early days, the PDF [5] database was available on 3 × 5 inch card sets and microfiche sets, with accompanying Alphabetic, Hanawalt, and Fink Search Manuals. Periodically several sets are re-edited and published in bound volumes. A subset of the PDF database was made available for search/match on magnetic tape. Many intermediate editorial and technical procedures were used to maintain quality, accuracy, and completeness of the database. In 1976, the JCPDS recognized the need to convert to a computer data base [6,7]. More details of the computerization process will be described below.

Powder diffraction patterns are compiled from journals, the ICDD Grant-in-aid Program, other grants and scientific contributions. The patterns are edited for correctness, and reviewed for quality and uniqueness by various experts of the field. Subfile mark assignment was also conducted by the editors. The careful editorial process assures that the ICDD maintains the highest standards for accuracy and quality of its database [8].

The PDF currently (2001 release) consists of 51 sets of data and 136 895 patterns (over 87 500 experimental and 49 000 patterns calculated from the ICSD (ICSD is the Inorganic Crystal Structure Database maintained by Fachsinformationzentrum (FIZ) in Karlsruhe and NIST). The file covers ceramic mineral, metal/alloy, organic and other inorganic crystalline materials. The PDF is subdivided into various subfiles, such as inorganic, mineral, organic, metal/alloy, common phases, ICSD, forensic, education, zeolite, explosive, superconductors, cement, corrosion, polymer, detergent, pigment, pharmaceutical, ceramics, and a separate subfile for the NBS patterns.

## 3. Collaborative Projects of the JCPDS-ICDD Research Associateship

The primary mission of the JCPDS-ICDD Research Associateship was to produce standard reference x-ray powder diffraction patterns for phase identification. Several additional research projects which were important for improvement of the quality of patterns and methodology were also identified and conducted under the supervision of the NBS scientific staff. These projects included the development of computer software for data collection and reduction, software to aid the powder pattern evaluation and editorial process, and quantitative phase analysis. Another area of important research area was the development of standard reference materials (SRM™) for instrument calibration and quantitative phase analysis.

### 3.1 Preparation of Standard Reference Patterns

These NBS standard patterns were produced using reproducible, controlled conditions of specimen preparation, and the best recording methods and instrumentation available. The method of producing these patterns has been described in detail by McMurdie et al. [9]. The consistently high quality of these patterns has been an integral part of the Powder Diffraction File (PDF). The directive of the JCPDS Associateship at NBS to prepare accurate data for the important inorganic substances has resulted in upgrading patterns already represented in the PDF as well as the addition of many new patterns from substances for which no data were available. Table 1 is an example of a NBS produced pattern for Au (PDF 4-784) prepared in year 1953. After all these years, it is still a sought-after standard, and is the only pattern for this material. The high quality of the data has shown to withstand the test of time.

The patterns prepared by the NBS Associateship were used for various applications. For example, they have often been singled out for reference when testing new methods involving x-ray diffraction. Also, as a final step in the alignment of any x-ray powder diffraction instrument, it is a common practice to record the pattern of some common materials, e.g., SiO_2_ (quartz), Al_2_O_3_ (corundum) etc., and to compare them against the reference patterns produced by the Associateship. In later years, with the introduction of computer-controlled experimental systems, and with the computer-based search-match procedures, this testing became more intensive. During the development of computer unit cell indexing routines, it was further recognized that only high quality data such as the Associateship patterns would consistently yield correct results.

Because of the extraordinary quality of the patterns produced by the Associateship, they have been collected under a single cover known as the NBS Patterns. This collection contains patterns for many compounds commonly encountered in scientific and industrial studies. Although these patterns have appeared in Circulars and Monographs published by the NBS, several of these publications were out of print at the time the NBS Patterns Book appeared. Also, earlier patterns have been revised as new techniques became available. A minifile called ‘NBS’ subfile was created in the PDF database, and these patterns also served as a minisearch file along with a specific search manual. The NBS patterns have also been used as an excellent teaching tool in the use of powder diffraction methods for compound identification. (The full PDF with a large number of patterns often led the beginners into many confusing pattern similarities.) These data cover many common laboratory chemicals and minerals so that in some cases identifications of common materials can be made quickly without a search through the full data collection. Teachers in classrooms often have found this minifile useful because most of the materials reported are readily available for classroom experiments.

In order to obtain a basis for an approximate quantitative analysis of mixtures, a comparative strength of peak height intensities from different materials is necessary. In addition to the crystallographic data, *d*-spacings, intensity values of the diffraction peaks, references, sample preparation procedure, another piece of information which is reported for a large number of patterns prepared by the Associateship is the reference intensity ratio (RIR). RIR is an instrument-independent constant for use in the quantitative phase analysis by the internal standard method [9a,^10^]. These constants can be measured or calculated. The choice of the best material to use as a standard was made by cooperations between NBS, Laboratorium voor Technische Natuurkunde, and Technisch Physische Dienst, and the JCPDS Research Associateship. A small particle size material corundum, *α*-Al_2_O_3_, was chosen as the internal standard for preparing the reference intensity ratio (RIR). When the reference standard is corundum, RIR is known as the *I*/*I*_c_. Recommended methods for accurate measurement of RIR constants were discussed by Hubbard and Snyder [10].

### 3.2 Automation of Data Collection and Reduction

The production of standard x-ray diffraction patterns at NBS imposed special requirements and specific demands in the automated data collection and data processing. The data collection system AUTO [11] and the data processing system POWDER-PATTERN [12], which was a flexible and an interactive data processing system, were developed in the early eighties. The POWDER-PATTERN suite consisted of a number of independent programs. These independent programs include PATTERN (locate peaks by smoothing data and calculating the second derivative), CALIBRATE (correct the 2*θ* values using internal and/or external calibration parameters), PLOT (perform both hardcopy and interactive plots), and PROFILE REFINEMENT (refined peak positions and their intensities). This software suite was critical for the Associateship members to generate high quality x-ray diffraction patterns. Many requests have been received from outside laboratories concerning the availability of these software products.

### 3.3 Development of Standard Reference Materials (SRM)

Based on the NBS/JCPDS collaborations, several standard reference materials (SRMs) were developed for powder x-ray diffraction measurements [13–15]. Among various SRMs developed by C. R. Hubbard and his coworkers, two were developed specifically to improve the quality of the PDF data. SRM 640 (640a and 640b were renewals) [14,15] were high-purity silicon powders that were prepared from electronic grade float-zone silicon boules. Recently SRM640c was certified by J. P. Cline et al. [16]. This standard was also prepared from ultra high purity, intrinsic silicon boules that were crushed and jet milled. The certification was conducted using a diffractometer built at NIST for that purpose and analyzed using a *Fundamental Parameter Approach* convolution algorithm [17]. SRM 675 is synthetic fluorophlogopite mica with initial diffraction peaks at much lower 2*θ* than those from Si [18]. They were used to correct for shifts of peak positions arising from sample, instrument, and physical aberrations. SRM 675 is intended for use as a low angle (large *d*-spacing) standard and is best used as pressed samples having a high degree of preferred orientation in which the 00l reflections have significant intensity. Both these SRMs were *d*-spacing standards which can be used as external or internal calibrants and are in high demand. External and internal standard calibration are important procedures for achieving high accuracy in x-ray powder diffraction studies. The theoretical basis as well as procedures for obtaining calibration curves, methods and examples of selecting SRMs, and procedures of sample preparation with these standards are described by McMurdie et al. [9] and Wong-Ng and Hubbard [19]. Other standards produced at NBS by Hubbard and colleagues during the 1980s include SRM660 (LaB_6_) for profile calibration, SRM 674 (a suite of 5 RIR calibrants consisting of corundum, fluorite, alumina, rutile, wurtzite), and SRM 1878 (respirable quartz).

Today a large collection of SRMs is available for powder x-ray diffractometry. Further development of new SRMs as well as recertification of existing ones continues under the leadership of J. P. Cline. He has developed a new SRM 1976 (alumina plate) and recertified three (SRM 660a, SRM 640c, and SRM 1878a) whose stock was exhausted. Their purpose, cost, and further information on these SRMs are available in the SRM office of NIST. Further information can be obtained via the Website (http://www.nist.gov/srm).

### 3.4 Computerization of the ICDD Powder Diffraction Database

Computerization of the PDF has been a significant undertaking, beginning in 1979 and substantially complete in 1985 [6,7]. The main goal of this undertaking were:

1. To enhance the quality of the PDF,
2. To increase the efficiency of the data entry, data editing, data review and record keeping.
3. To simplify the production efforts by eliminating the need to maintain the database in two separate forms.
4. To eliminate problems such as incompleteness of data and rounding of *d*-spacings on the PDF tape.
5. To have a more flexible and informative database.
6. To enhance the assessment of quality of the data.
7. To evaluate all patterns with computer-assisted methods.

In 1976, G. J. McCarthy, Chairman of the Technical Committee of ICDD, established a task group, which subsequently became the Data Base Subcommittee (chaired by C. R. Hubbard). A proposal which entailed a five year plan of creating computer code, designing and testing a prototype database, and reevaluating the historical data of Sets 1–32 was submitted by this subcommittee and was approved by the ICDD Board of Directors. The computerization was carried out cooperatively by both the ICDD and NBS staff. The NBS staff provided the technical guidance for the entry process and wrote the key computer programs which were necessary for the creation and critical evaluation of the computer database. Members of the ICDD staff performed the critical review, developed editorial procedures, and provided additional data entry. Work on the proposal began at NBS with the design of the database format (structure of the database), selection of data items to be included, and development of a computer program to evaluate the data entries. The format selected for the PDF database was that of NBS CRYSTAL DATA [20] developed by NBS Crystal Data Center [21] and expanded to include powder pattern information. A computer program, known as the NBS*AIDS80 [22], was designed to build and evaluate a database entry. The later version of the program is known as the NBS*AIDS83.

If NBS*AIDS80 finds a data or format inconsistency (“error”), then the data must be corrected and reprocessed. Patterns with unresolvable errors were added to a list of questionable phases for remeasurement. The program also gives warnings of possible errors in the data, each of which are reviewed, and, if necessary, editorial changes are made. Throughout the years, this program has been updated extensively to meet the need of a variety of new classes of material, and the state-of-the-art computer database and file structure. It still remains as the key editorial program for producing the PDF. Because of this sophisticated computer program, the review process utilized today is considerably more stringent than in former years [8]. Because of the important impact of this program on the evaluation of diffraction data as well as deriving various ICDD products, a detailed description of the NBS*AIDS83 program is given as an appendix.

The full-scale project of computerization and critical evaluation of PDF data Sets 1–32 was initiated in the beginning of 1982. All data entering the PDF beginning with data for Set 33 were not part of the historical review but were evaluated and computerized during the editorial and publication process. Since 1984, products such as data books and data cards (discontinued to date) have been produced directly from the PDF database. Today, all PDF products are generated directly from the database, including a recently released relational database product. Further information about the ICDD products can be located at the WEBSITE: http://www.icdd.com.

### 3.5 Compact Disk Read Only Memory (CD-ROM) System for Data Storage

Another development of the ICDD database concerns data storage using a CD-ROM disk. In the mid-eighties, there was increasing use of the PDF in computer readable form, but the limited amount of disk space available on most commercial powder diffractometer systems limited the use to a small subset of the total PDF. A product proposal was submitted by W. Wong-Ng and C. Hubbard to the JCPDS-ICDD Board of Director in the early eighties, followed by several presentations concerning the advantages and various product opportunities of using the state-of-the-art CD-ROM technology. The availability of low-cost CD-ROM systems offered an attractive alternative to conventional disk media [23]. Under the guidance of R. Jenkins, then ICDD Principal Scientist, the concept of a low-cost Personal Computer/CD-ROM system, “PC-PDF”, having a total available storage of 550 MB, was developed in the late eighties. A variety of search strategies were developed that are based on PDF numbers, chemistry, strongest *d*-spacings, etc. By use of optimum packing and access algorithms, the various searches operated at speeds highly convenient to the user. Currently, the CD-ROM products are the most popular products by ICDD.

## 4. Advanced Ceramic Reference Pattern Program at NBS (1986–1989)

With the development of standards, software, automated systems and numerous publications of data and methods, more and more laboratories became capable of measuring and analyzing high quality reference patterns. Thus, the mission of the ICDD Research Associate Program was believed to be accomplished in 1986, and part of the staff of the ICDD Associateship was transferred to the ICDD Headquarters. Following this success, a 3-year program at NBS to produce high quality x-ray patterns of important ceramic phases was established. This program was administered by C. R. Hubbard and A. L. Dragoo, with W. Wong-Ng was in charge of the day-to-day operation. The project was directed toward the production of x-ray diffraction data for important ceramic phases, which might find applications in engine components, cutting tools, etc. This project which was an integral part of the program at NBS to develop measurement methods and data to further the manufacture and use of ceramic materials, included a comprehensive review and upgrade of data for selected borides, carbides, silicides, nitrides, oxynitrides, selenides, tellurides and oxides. Samples for x-ray characterization were obtained through collaboration with other research laboratories, and by synthesis at NBS. For example, cooperation with the Phase Diagram Project, jointly sponsored by the American Ceramic Society and NBS, was initiated to identify new phases of interest, to synthesize selected new phases, and to develop improved editorial procedures for both the PDF and the Phase Diagram Database [24].

## 5. ICDD Grants-in-Aid Project

At the conclusion of the Advanced Ceramics Program, further collaborations continued through the ICDD Grants-in-Aid Program. This ongoing project compliments the NIST existing program of phase equilibria of electronic materials. In recent years, several important classes of electronic materials have had a large impact on the relevant industries. The characterization of high *T*_c_ materials has been an important activity of the superconductor community in the past 15 years. Many high *T*_c_ superconductor phases were reported in the literature, including new structure types as well as single phases and solid solutions isomorphous to known structures. The development of microwave materials for wireless communications has been steadfast. There has also been intense development of magnetic (i.e., magneto resistive materials) and ferroelectric materials for thin film applications. All of these fields are developing rapidly. The availability of high-quality powder patterns of carefully-prepared and well-characterized materials will meet the expressed needs of researchers in these fields.

In collaboration with J. Kaduk of BP-Amoco Research Center, patterns were analyzed using the Rietveld refinement technique [25]. Data processing was performed using the General Structure Analysis System (GSAS) [26]. The peaks are located by LeBail extractions of the raw patterns, using pseudo-Voigt profile functions. The internal standard results in corrections for systematic errors, principally sample displacement and transparency. Crystallographic cells, if unknown, were determined using the ITO [27] and/or Boultif & Louer [28] indexing programs. The reported lattice parameters were determined from profile fits. Refinements of the crystal structures are used to analyze and confirm details of the space group symmetry.

## 6. Further Collaborations

Fruitful collaborations between NIST and ICDD will continue for many years to come. In addition to the Grants-in-aid projects for the preparation of standard reference x-ray patterns for inclusion in the PDF, several NIST staff members are currently actively involved in the ICDD Scientific Subcommittee activities. Howard McMurdie continues to be the editor for the patterns of inorganic materials. He also works on the American Ceramic Society’s (ACS) compilation of phase diagrams. Collaborations between the NIST, ICDD and Ceramics Society database efforts have also been planned. For example, linkage of their respective databases would provide the user with the ability to search multiple databases in an integrated fashion. This cross-search capability will have an important impact on material characterization and material design.

## 7. Associateship Publications

Since the 1950s when the Research Associateship was established, it has produced nearly 2000 high quality standard reference patterns. The NBS patterns were published in NBS Circular 539 (10 volumes) and NBS Monographs (a total of 31 volumes), PDF database, NBS circulars, as well as the early issues of the journal Powder Diffraction. The following is a list of the principal publications from the Research Associateship program.

1. H. E. Swanson and E. Tatge, J. Res. Natl. Bur. Stand. (U.S.) **46** (4), 318 (1951), Data on 8 phases.
2. H. E. Swanson and E. Tatge, NBS Circular 539, Vol. 1 (1953), data for 54 inorganic substances.
3. H. E. Swanson and R. K. Fuyat, NBS Circular 539, Vol. 2 (1953), data for 30 inorganic substances.
4. H. E. Swanson, R. K. Fuyat, and G. M. Ugrinic, NBS Circular 539, Vol. 3 (1954), data for 34 inorganic substances.
5. H. E. Swanson, R. K. Fuyat, and G. M. Ugrinic, NBS Circular 539, Vol. 4 (1955), data for 42 inorganic substances.
6. H. E. Swanson, N. T. Gilfrich, and G. M. Ugrinic, NBS Circular 539, Vol. 5 (1955), data for 45 inorganic substances.
7. H. E. Swanson, N. T. Gilfrich, and M. I. Cook, NBS Circular 539, Vol. 6 (1956), data for 44 inorganic substances.
8. H. E. Swanson, N. T. Gilfrich, and M. I. Cook, NBS Circular 539, Vol. 7 (1957), data on 53 substances.
9. H. E. Swanson, N. T. Gilfrich, M. I. Cook, R. Stinchfield, and P. C. Parks, NBS Circular 539, Vol. 8 (1959), data for 61 substances.
10. H. E. Swanson, M. I. Cook, T. Isaacs, and E. H. Evans, NBS Circular 539, Vol. 9 (1960), data for 43 substances.
11. H. E. Swanson, M. I. Cook, E. H. Evans, and J. H. deGroot, NBS Circular 539, Vol. 10 (1960), data for 40 substances.
12. H. E. Swanson, M. C. Morris, R. P. Stinchfield, and E. H. Evans, NBS Monograph 25, Sec. 1 (1962), data for 46 substances.
13. H. E. Swanson, M. C. Morris, R. P. Stinchfield, and E. H. Evans, NBS Monograph 25, Sec. 2 (1963), data for 37 substances.
14. H. E. Swanson, M. C. Morris, E. H. Evans, and L. Ulmer, NBS Monograph 25, Sec. 3 (1964), data for 51 substances.
15. H. E. Swanson, M. C. Morris, and E. H. Evans, NBS Monograph 25, Sec. 4 (1966), data for 103 substances.
16. H. E. Swanson, H. F. McMurdie, M. C. Morris, and E. H. Evans, NBS Monograph 25, Sec. 5 (1967), data for 80 substances.
17. H. E. Swanson, H. F. McMurdie, M. C. Morris, and E. H. Evans, NBS Monograph 25, Sec. 6 (1968), data for 60 substances.
18. H. E. Swanson, H. F. McMurdie, M. C. Morris, and E. H. Evans, NBS Monograph 25, Sec.7 (1969), data for 81 substances.
19. H. E. Swanson, H. F. McMurdie, M. C. Morris, and E. H. Evans, NBS Monograph 25, Sec. 8 (1970), data for 81 substances.
20. H. E. Swanson, H. F. McMurdie, M. C. Morris, E. H. Evans, and B. Paretzkin, NBS Monograph 25, Sec. 9 (1971), data for 63 substances.
21. H. E. Swanson, H. F. McMurdie, M. C. Morris, E. H. Evans, and B. Paretzkin, NBS Monograph 25, Sec. 10 (1972), data for 84 substances.
22. H. E. Swanson, H. F. McMurdie, M. C. Morris, E. H. Evans, and B. Paretzkin, NBS Monograph 25, Sec. 11 (1974), data for 70 substances.
23. H. F. McMurdie, M. C. Morris, E. H. Evans, B. Paretzkin, J. H. deGroot, C. R. Hubbard, and S. J. Carmel, NBS Monograph 25, Sec. 12 (1975), data for 57 substances.
24. M. C. Morris, H. F. McMurdie, E. H. Evans, B. Paretzkin, J. H. deGroot, C. R. Hubbard, and S. J. Carmel, NBS Monograph 25, Sec. 13 (1976), data for 58 substances.
25. M. C. Morris, H. F. McMurdie, E. H. Evans, B. Paretzkin, J. H. deGroot, R. Newberry, C. R. Hubbard, and S. J. Carmel, NBS Monograph 25, Sec. 14 (1977), data for 68 substances.
26. M. C. Morris, H. F. McMurdie, E. H. Evans, B. Paretzkin, J. H. deGroot, B. S. Weeks, R. J. Newberry, C. R. Hubbard, and S. J. Carmel, NBS Monograph 25, Sec. 15 (1978), data for 112 substances.
27. M. C. Morris, H. F. McMurdie, E. H. Evans, B. Paretzkin, J. H. deGroot, C. R. Hubbard, and S. J. Carmel, NBS Monograph 25, Sec. 16 (1979), data for 86 substances.
28. M. C. Morris, H. F. McMurdie, E. H. Evans, B. Paretzkin, C. R. Hubbard, and S. J. Carmel, NBS Monograph 25, Sec. 17 (1980), data for 54 substances.
29. M. C. Morris, H. F. McMurdie, E. H. Evans, B. Paretzkin, H. S. Parker, N. C. Panagiotopoulos, and C. R. Hubbard, NBS Monograph 25, Sec. 18 (1981), data for 58 substances.
30. M. C. Morris, H. F. McMurdie, E. H. Evans, B. Paretzkin, H. Parker, N. P. Pyrros, and C. R. Hubbard, NBS Monograph 25, Sec. 19 (1982), data for 51 substances.
31. M. C. Morris, H. F. McMurdie, E. H. Evans, B. Paretzkin, H. S. Parker, N. P. Pyrros, and C. R. Hubbard, NBS Monograph 25, Sec. 20 (1984), data for 71 substances.
32. M. C. Morris, H. F. McMurdie, E. H. Evans, B. Paretzkin, H. S. Parker, W. Wong-Ng, D. M. Gladhill, and C. R. Hubbard, NBS Monograph 25, Sec. 21 (1985), data for 92 substances.
33. H. F. McMurdie, M. C. Morris, E. H. Evans, B. Paretzkin, W. Wong-Ng, L. Ettlinger, and C. R. Hubbard, Powder Diffraction **1** (1), 77 (1986), data on 20 phases.
34. H. F. McMurdie, M. C. Morris, E. H. Evans, B. Paretzkin, W. Wong-Ng, L. Ettlinger, and C. R. Hubbard, Powder Diffraction **1** (2), 64 (1986), data on 20 phases.
35. H. F. McMurdie, M. C. Morris, E. H. Evans, B. Paretzkin, W. Wong-Ng, and C. R. Hubbard, Powder Diffraction **1** (3), 265 (1986), data on 15 phases.
36. H. F. McMurdie, M. C. Morris, E. H. Evans, B. Paretzkin, W. Wong-Ng, Y. Zhang, and C. R. Hubbard, Powder Diffraction **1** (4), 334 (1986), data on 17 phases.
37. H. F. McMurdie, M. C. Morris, E. H. Evans, B. Paretzkin, W. Wong-Ng, Y. Zhang, and C. R. Hubbard, Powder Diffraction **2** (1), 41 (1987), data on 17 phases.
38. W. Wong-Ng, H. F. McMurdie, B. Paretzkin, C. R. Hubbard, A. L. Dragoo, and J. M. Stewart, Powder Diffraction **2** (2), 106 (1987), data on 15 phases.
39. W. Wong-Ng, H. F. McMurdie, B. Paretzkin, Y. Zhang, K. L. Davis, C. R. Hubbard, A. L. Dragoo, and J. M. Stewart, Powder Diffraction **2** (3), 191 (1987), data on 16 phases.
40. W. Wong-Ng, H. F. McMurdie, B. Paretzkin, Y. Zhang, K. L. Davis, C. R. Hubbard, A. L. Dragoo, and J. M. Stewart, Powder Diffraction **2** (4), 257 (1987), data on 15 phases.
41. W. Wong-Ng, H. F. McMurdie, B. Paretzkin, Y. Zhang, C. R. Hubbard, A. L. Dragoo, and J. M. Stewart, Powder Diffraction **3** (1), 47 (1988), data on 15 phases.
42. W. Wong-Ng, H. F. McMurdie, B. Paretzkin, C. R. Hubbard, and A. L. Dragoo, Powder Diffraction **3** (2), 113 (1988), date on 14 phases.
43. W. Wong-Ng, H. F. McMurdie, B. Paretzkin, M. A. Kuchinski, and A. L. Dragoo, Powder Diffraction **3** (3), 179 (1988), data on 14 phases.
44. W. Wong-Ng, H. F. McMurdie, B. Paretzkin, M. A. Kuchinski, and A. L. Dragoo, Powder Diffraction **3** (4), 247 (1988), data on 14 phases.
45. W. Wong-Ng, H. F. McMurdie, B. Paretzkin, M. A. Kuchinski, and A. L. Dragoo, Powder Diffraction **4** (1), 40 (1989), data on 14 phases.
46. W. Wong-Ng, H. F. McMurdie, B. Paretzkin, M. A. Kuchinski, and A.L. Dragoo, Powder Diffraction **4** (2), 106 (1989), data on 15 phases.

## Figures and Tables

**Table 1:** JCPDS/NBS powder diffraction pattern of Au (4-784), reproduced with the permission of the ICDD

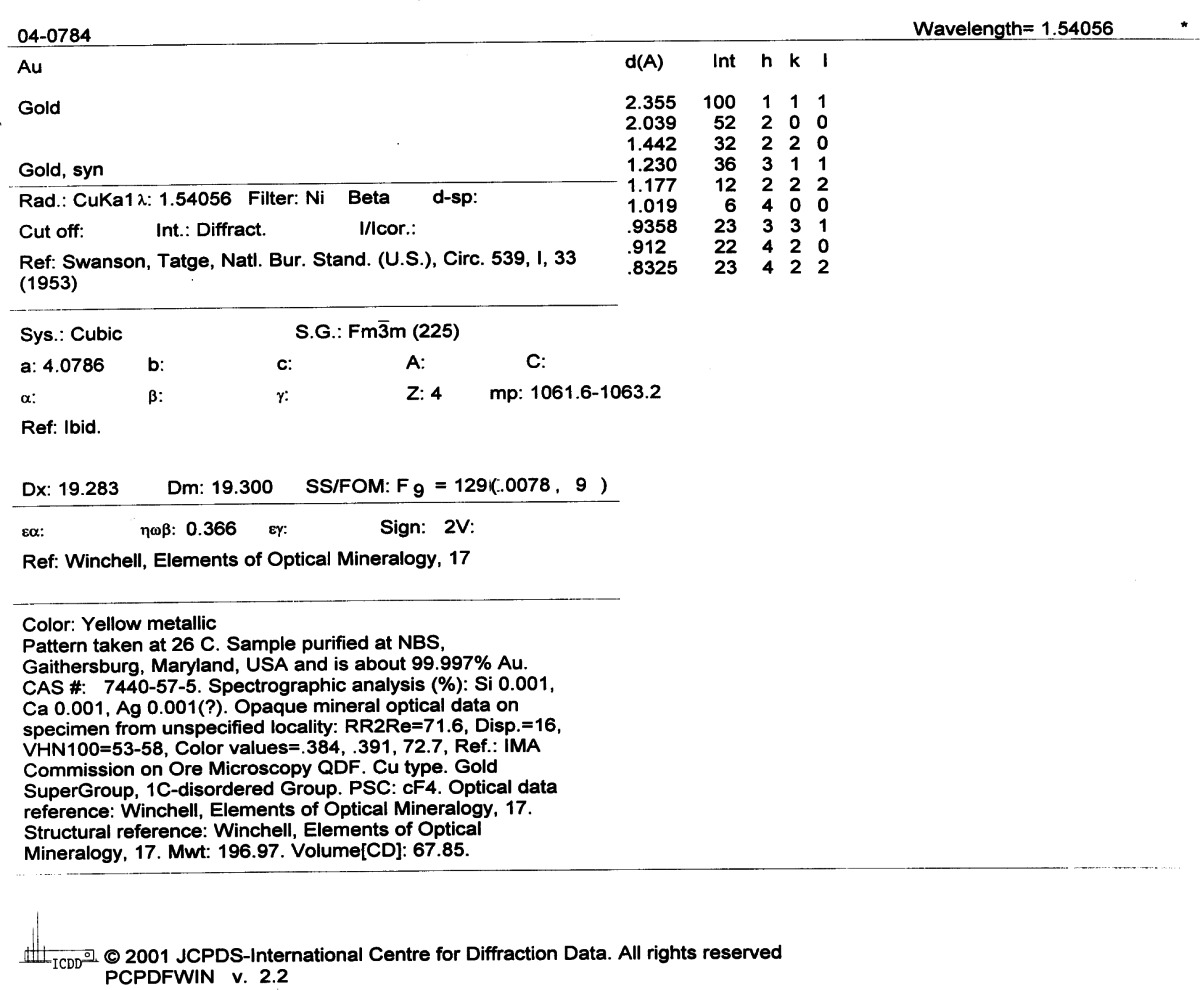

**Table 2:** Data Records and labeling in NBS*AIDS83

No.	Type	Max^a^	Contents
1	1	2	Original lattice parameters
2	2	2	Author’s standard deviations of lattice parameters
3	3	1	Author’s space group, *Z*, density
4	4	1	Crystal Data space group, *Z*, density
5	5	5	Subfile designations, mineral and zeolite groups, CAS No.
6	6	5	Compound name
7	7	5	Chemical formula
8	8	1	Empirical formula
9	9	10	Literature references
10	A	1	Pearson symbol, structure type
11	B	20	Comments
12	C	1	Transformation matrix: initial to Crystal Data cell
13	D	1	Reduced cell
14	E	1	Crystal Data cell
15	F	1	Information on data collection (e.g., wavelength)
16	G	1	Further information on data collection (e.g., quality marks)
17	H	1	Space group extinction conditions
18	I	68	Powder diffraction data (*d* or 2*θ*, relative intensities, *hkl*, editorial flags)
19	J	5	Internal update and revision information
20	+	1	Information for Hanawalt and Fink indices
21	*	1	Information for Max-*d* index
22	K^b^	1	Processing history and entry-end record

a Maximum number of 80 character records for a given record type.

b The K record is the final record of any entry, following the plus and star records (if present).

**Table 3:** Selected functions performed by NBS*AIDS83. The program is multifunctional as it evaluates data, transforms information to standard settings, and generates key derivative data

No.	Function
1.	Calculation of the reduced cell
2.	Transformation of author’s cell to conventional Crystal Data cell
3.	Transformation of authors’ space group to Crystal Data setting
4.	Verification of space group with allowed symbols
5.	Determination of metric lattice symmetry
6.	Comparison of metric symmetry with crystal symmetry
7.	Formula syntax checking
8.	Format of the chemical name
9.	Comparison of chemical name with formula for consistency
10.	Generation of empirical formula
11.	Generation of Pearson symbol
12.	Comparison of measured, calculated and estimated densities
13.	Refinement of *d*-spacings
14.	Confirmation of indexing with space group extinction rules
15.	Calculation of the Smith-Snyder and de Wolff figures-of-merit
16.	Assignment of the PDF quality mark
17.	Reference syntax checking
18.	Validation of Journal CODEN against list
19.	Validation of editorial flags
20.	Generation of error and warning flags when problems are detected

## 8. Appendix A. A Program for Data Evaluation and Database Building—NBS*AIDS83

### 8.1 Introduction

The program NBS*AIDS83 has three basic functions: 1) data evaluation and database building, 2) data transformation to standard settings (e.g., crystal data cell and space group), and 3) derivative data generation (e.g., reduced cell, Pearson’s symbol). Three distribution databases are built with this program—NIST Crystal Data, International Centre for Diffraction Data PDF-2 and the Electron Diffraction Database. The program, research applications, and detailed information on many items in common in the databases have been described in NBS Technical Notes [22].

NBS*AIDS83 is a large, dynamic and evolving program with over 17 000 statements and 100 subroutines. Over the years, many scientists have made contributions to the program. Many man-years have been invested in creating the software and in devising the theory underlying many of the routines. Because numerous commercial computer programs use the output from NBS*AIDS83, every effort has been made during program revisions and expansions to keep the formats for all existing data items constant.

The NBS*AIDS 83 program, or its precursor which was designed and written in the early 1970s for Crystal Data, has been in continual use for about 30 years. The program is used by the scientific community in several ways. First, editors at NIST, the ICDD, and at other data centers have used this workhorse program as a tool to create hundreds of thousands of evaluated database entries. Second, a research version of the program has been distributed to the scientific community, which has served to improve the quality of experimental data at the source. Finally, selected segments of the program have been incorporated in other widely used distribution programs such as the indexing program (DICVOL91) of Boultif and Louer [28] and the Structure Tidy program of Gelato and Parthé [29]. A key reason for its success and reliability, and that the program has been used for a long period of time, by so many people, on so much data, in so many situations, is the extensive effort in code design, database design and validation of its functions.

The program has played a critical role in the scientific and commercial success of the distribution databases. This is especially true for the PDF-2 and its derivatives, which are universally used by the powder diffraction community. All three databases cited above are associated with commercial instruments that collect diffraction data. Thus, the collection of new data is fully integrated with the use of existing data as, for example, in the identification of unknown compounds. Finally, the transformation routines in NBS*AIDS83 have made it possible for standard crystallographic cell and space group settings—devised by NIST—to be used by scientists world-wide.

### 8.2 Discussion

The overall job of the program is to build a database entry of evaluated data for NIST Crystal Data [18] or the Powder Diffraction File—PDF-2. The program features include: a) the same program is used to build both databases. The fact that the software is used by more than one independent data center has played a central role in building comprehensive and robust computer code; b) all the data for an entry are evaluated as a unit at the same time. This feature permits easy and efficient editing of the entry; and c) the data evaluation and database building functions are integrated.

Both databases include chemical, crystallographic and bibliographic data for all classes of crystalline compounds—inorganics, organics, minerals, metals, alloys, ceramics, intermetallics etc. A database entry includes data for one compound and includes such data as: chemical name and formula, lattice parameters, space group, density, structure type, literature reference and comments. The main difference between NIST Crystal Data and the PDF-2 is that the latter includes powder diffraction data. Overall database features include: a) all data parameters have been carefully defined, formatted and documented. By doing this, the full power of computing can be applied to data evaluation, extraction, and utilization; b) the ASCII character set is used for all data items. This has made the database highly transportable; c) the entry output of a computer run can be used as the input for a later run. This feature makes it possible to update the entire database concurrently with a revision of the NBS*AIDS83 computer program.

#### 8.2.1 Database Specifications

The information set for each entry in the database is divided into 22 record types as summarized in Table 2. Each entry must begin with record type 1, and the entry ends with the record type K. Each record type contains information that is stored in units of 80 ASCII characters, and some record types contain multiple units. Table 2 gives a summary of record types, and the maximum number of units allowed for each type. As indicated above, all data parameters in the database follow rigorous rules. Detailed database specifications for each record type are given in the NBS Technical Note 1141 [22] and in the internal documentation for the International Centre for Diffraction Data’s current version.

#### 8.2.2 Program Functions

The program carries out three basic types of functions: 1) data transformation and standardization; 2) the generation of derivative data; and 3) data evaluation. The computer code consists of over 100 subroutines. In general the different subroutines are responsible for different functions or subfunction. In Table 3, a summary of the various functions is given.

##### 8.2.2.1 Transformation and Standardization

The unit cell and space group data are transformed to the conventional crystal data settings. By doing this, one can identify unknown materials against the database on the basis of the determinative ratios (i.e., ratios of specified cell edges). Furthermore, data on the same material that have been reported on the basis of different unit cells will be transformed to the same setting. The Crystal Data Center and program have played an important role in the collection and reporting of crystallographic data. The conventional crystal data cell—or a closely related cell—calculated by the program is now widely accepted by the crystallographic community for the reporting of such data in the literature. The use of such standard cells has improved the quality and reliability of the data reported in the original literature.

##### 8.2.2.2 Generation of Derivative Data

An important function of the program is the generation of key derivative data that would be of value to the research scientist. This data is obtained via specially designed software routines. Examples of derivative data that are routinely used for a variety of purposes include the reduced cell [22a], the empirical formula and Pearson’s symbol [22b]. The first two derivative parameters serve as the basic data for a powerful method for the identification and characterization of compounds. The third, Pearson’s symbol, is widely used for determining structurally related metals and intermetallics.

##### 8.2.2.3 Data Evaluation

The data within an entry is evaluated as a unit. This strategy permits the database editors to evaluate readily the correctness and consistency of the chemical and crystallographic data on a given crystalline compound. Many types of checks are made which result in error and warning messages when problems are encountered. The general categories of checks on the data include a syntax-format check, a legality check, and scientific-sense check. The following examples illustrate the nature of the checks. Formulas are checked to verify that they conform to a rigorous syntax and format so that empirical formulas can be generated. The original and transformed space group symbols are checked against a list of acceptable symbols to assure that they are valid. The crystal symmetry is flagged if the metric exceeds the crystal symmetry. A warning message appears when the authors density and program-calculated density are in disagreement. Ideally the editors correct data parameters so that the all error and most warning messages disappear when the data are reprocessed.

#### 8.2.3 Advantages

After using the program continuously for more than 20 years, certain advantages to the software and the approach used by the data centers have become clear. The advantages are related to program reliability, database quality and external impact.

##### 8.2.3.1 Program Reliability

For a generation, the program has been used by two independent data centers for the production of their respective databases. Therefore, the code has evolved into a highly debugged and valuable tool as many editors have used the software on all types of data, over an extended period of time. In respond to editorial suggestions and requirements, revisions have been periodically made to strengthen the program. The development and use of the program represent a truly cooperative effort by many scientists with respect to code creation and to rules and conventions on how parameters should be formatted, transformed, and included in the database.

##### 8.2.3.2 Database Quality

The program has lead to high quality scientific databases—NIST Crystal Data and the Powder Diffraction File—PDF-2. Of special note is the fact that the PDF-2 is one of the most widely used and successful of the scientific databases. The program has permitted these databases to be highly reliable and searchable as every parameter is carefully defined and evaluated, and important derived parameter are generated by mathematical algorithm following rigorous rules. The program has allowed human and computer editing to work in a synergistic manner.

##### 8.2.3.3 External Impact

A research version of the program [1] has been made available to the scientific community so that individual scientists can evaluate and transform their own experimental data. This has had an important impact. For example, the cell and space group transformation routines in the software have made it possible for the experimentalists to use standard settings for the collection and reporting of their data. Furthermore, the research version of the software has permitted scientists to evaluate their data prior to publication thereby preventing errors in the literature. Finally selected subroutines of the code have been incorporated into other widely distributed scientific software packages.

### 8.3 The Future

The NBS*AIDS83 program has served the database editors well for many years. It will undoubtedly continue to be used. However, with respect to the times, the program now has severe limitations. For example, the editors for the databases need many additional tools that are now available due to theoretical and computational advances. One critical tool is a modern database management system. Other tools incorporate other scientific evaluation programs that can be used in conjunction with the database [30–34]. Consequently, this is an appropriate time for a comprehensive change.

The principles and software code for building a new system are all known and in place. However, putting them all together into a modern evaluation system that can be used by multiple editors to build more than one scientific database is the challenge. The importance of meeting this challenge cannot be overemphasized!
